# Determination of Optimal Measurement Points for Calibration Equations—Examples by RH Sensors

**DOI:** 10.3390/s19051213

**Published:** 2019-03-09

**Authors:** Hsuan-Yu Chen, Chiachung Chen

**Affiliations:** 1 Department of Materials Science and Engineering, University of California, San Diego, CA 92093, USA; wakaharu37@gmail.com; 2 Department of Bio-Industrial Mechatronics Engineering, National ChungHsing University, Taichung 40227, Taiwan

**Keywords:** calibration points, saturated salt solutions, humidity sensors, measurement uncertainty

## Abstract

The calibration points for sensors must be selected carefully. This study uses accuracy and precision as the criteria to evaluate the required numbers of calibration points required. Two types of electric relative humidity (RH) sensors were used to illustrate the method and the standard RH environments were maintained using different saturated salt solutions. The best calibration equation is determined according to the *t*-value for the highest-order parameter and using the residual plots. Then, the estimated standard errors for the regression equation are used to determine the accuracy of the sensors. The combined uncertainties from the calibration equations for different calibration points for the different saturated salt solutions were then used to evaluate the precision of the sensors. The accuracy of the calibration equations is 0.8% RH for a resistive humidity sensor using 7 calibration points and 0.7% RH for a capacitance humidity sensor using 5 calibration points. The precision is less than 1.0% RH for a resistive sensor and less than 0.9% RH for a capacitive sensor. The method that this study proposed for the selection of calibration points can be applied to other sensors.

## 1. Introduction

The performance of sensors is key for modern industries. Accuracy and precision are the most important characteristics. Calibration ensures sensors’ performance. When a sensor is calibrated, the reference materials or reference environments must be specified. For a balance calibration, a standard scale is the reference materials. For temperature calibration, the triple point of ice-water or boiling matter is used to maintain the reference environment.

The experimental design for calibration must consider the following factors [1,2,3].

1. The number and the location of the calibration points.
2. The regression equations (linear, poly-nominal, non-linear).
3. The regression techniques.
4. The standard references and their uncertainties.

Betta [1] adopted minimizing the standard deviations for the regression curve coefficients or the standard deviation for the entire calibration curve to design an experiment to determine the number of calibration points, the number of repetitions, and the location of calibration points. Three types of sensor were used to demo the linear, quadratic and cubic calibration equations: a pressure transmitter, a platinum thermometer and E-Type thermocouple wires. The estimated confidence interval values were used to determine the validity of the regression equation. This method was extended to address calibration for complex measurement chains [2].

Hajiyev [3] noted the importance of the selection of the calibration points to ensure the accuracy of the calibration and the optimal selection of standard pressure setters and used an example to verify the method. A dispersion matrix, $\underset{D}{\to }$ of the estimated coefficients was defined and this matrix $\underset{D}{\to }$ was used as a scale of the error between the sensor and the reference instruments. Two criteria were used to evaluate the performance. The minimized sum of the diagonal elements of the matrix $\underset{D}{\to }$ is called the A-optimality criterion. The minimized of the generalized of determinant of the matrix $\underset{D}{\to }$ is called the D-optimality criterion. The optimal measurement points for the calibration of the differential pressure gages were determined using the A-optimality criterion [3] and the D-optimality criterion [4]. Khan et al. [5] used an inverse modeling technique with a critical neural network (ANN) to evaluate the order of the models and the calibration points. The root-mean-square error (RMSE) was used as the criterion.

Recently, modern regression has been used as an important role to express the quantitative relationship between independent and response variables for tests on a single regression coefficient [6,7,8,9]. This technique used to address calibration equations and the standard deviations of these calibration equations then served as the criteria to determine their accuracy [10,11].

The confidence band for the entire calibration curve or for each experimental point was used to evaluate the fit of calibration equations [1,2]. The concept of measurement uncertainty (MU) is widely used to represent the precision of calibration equations [12,13,14]. Statistical techniques can be used to evaluate the accuracy and precision of calibration equations that are obtained using different calibration points [15,16,17]. Humidity sensors that were calibrated using different saturated salt solutions were tested to illustrate the technique for the specification of optimal measurement points [18,19].

Humidity is very important for various industries. Many manufacturing and testing processes, such as those for food, chemicals, fuels and other products, require information about humidity [20]. Relative humidity (RH) is commonly used to express the humidity of moist air [21]. Electric hygrometers are the most commonly used sensors because they allow real-time measurement and are easily operated.

The key performance factors for an electrical RH meter are the accuracy, the precision, hysteresis and long-term stability. At high air humidity measurement, there is a problem with response time of the RH sensors in conventional methods. The solution for this problem for high air humidity measurement is to use an open capacitor with very low response time [22,23,24] and quartz crystals which compensate temperature drift. An environment with a standard humidity is required for calibration. Fixed-point humidity systems that use a number of points with a fixed relative humidity are used as a standard. A humidity environment is maintained using different saturated salt solutions. The points with a fixed relative humidity are certified using various saturated salt solutions [19]. When the air temperature, water temperature and air humidity reach an equilibrium state, constant humidity is maintained in the air space [19].

The RH value that is maintained by the salt solutions is of interest. Wexler and Hasegawa measured the relative humidity that is created by eight saturated salt solutions using the dew point method [25]. Greenspan [18] compiled RH data for 28 saturated salt solutions. The relationship between relative humidity and ambient temperature was expressed as a 3rd or 4th polynomial equation. Young [26] collected RH data for saturated salt solutions between 0 to 80 °C and plotted the relationship between relative humidity and temperature. The Organisation Internationale De Metrologies Legale (OIML) [19] determined the effect of temperature on the relative humidity of 11 saturated salt solutions and tabulated the result. Standard conditions, devices and the procedure for using the saturated salt solutions were detailed.

The range for the humidity measurement is from about 11% to 98% RH. Studies show that the number of fixed-point humidity references that are required for calibration is inconsistent. Lake et al. [27] used five salt solutions for calibration and found that the residuals for the linear calibration equation were distributed in a fixed pattern. Wadso [28] used four salt solutions to determine the RH that was generated in sorption balances. Duvernoy et al. [29] introduced seven salt solutions to generate the RH for a metrology laboratory. Bellhadj and Rouchou [30] recommended five salt solutions and two sulfuric acids to create the RH environment to calibrate a hygrometer.

There is inconsistency in the salt solutions that are specified by instrumentation companies and standard bodies. The Japanese Mechanical Society (JMS) specifies 9 salt solutions for the standard humidity environment [31]. The Japanese Industrial Standards Committee (JISC) recommends 4 salt solutions to maintain RH environment [32]. The Centre for Microcomputer Applications (CMA) company specifies 11 salt solutions [33]. Delta OHM use only 3 salt solutions [34]. The OMEGA company use 9 salt solutions [35]. TA instruments specifies 9 salt solutions [36] and Vaisala B.V. select 4 salt solutions [37]. These salt solutions are listed in Table 1.

Lu and Chen [17] calculated the uncertainty for humidity sensors that were calibrated using 10 saturated salt solutions for two types of humidity sensors. The study showed that a second-order polynomial calibration equation gave better performance than a linear equation. The measurement uncertainty is used as the criterion to determine the precision performance of sensors [38].

The number of standard relative humidity values for fixed-point humidity systems is limited by the number and type of salt solutions. The number of salt solutions that must be used to specify the calibration points for the calibration of RH sensors is a moot point. More salt solutions allow more calibration points for the calibration of RH sensors. However, using more salt solutions is time-consuming. This study determined the effect of the number and type of salt solutions on the calibration equations for two types of humidity sensors. The accuracy and precision were determined in order to verify the method for the choice of the optimal calibration points for sensor calibration.

## 2. Materials and Methods

### 2.1. Relative Humidity (RH) and Temperature Sensors

Resistive sensor (Shinyei THT-B141 sensor, Shinyei Kaisha Technology, Kobe, Japan) and capacitive sensor (Vaisala HMP-143A sensor, Vaisala Oyj, Helsinki, Finland) were used in this study. The specification of the sensors is listed in Table 2.

### 2.2. Saturated Salt Solutions

Eleven saturated salt solutions were used to maintain the relative humidity environment. These salt solutions are listed in Table 3.

### 2.3. Calibration of Sensors

The humidity probes for the resistive and capacitive sensors were calibrated using saturated salt solutions. A hydrostatic solution was produced in accordance with OIML R121 [19]. The salt was dissolved in pure water in a ratio such that 40–75% of the weighted sample remained in the solid state. These salt solutions were stored in containers.

The containers were placed in a temperature controller at an air temperature of 25 ± 0.2 °C. During the calibration process, humidity and temperature probes were placed within the container above the salt solutions. The preliminary study showed that an equilibrium state is established in 12 h so the calibration lasted 12 h to ensure that the humidity of the internal air had reached an equilibrium state. Experiments for each RH environment were repeated three times. The temperature was recorded and the standard humidity of the salt solutions was calculated using Greenspan’s equation [18].

### 2.4. Establish and Validate the Calibration Equation

**Figure 1:**
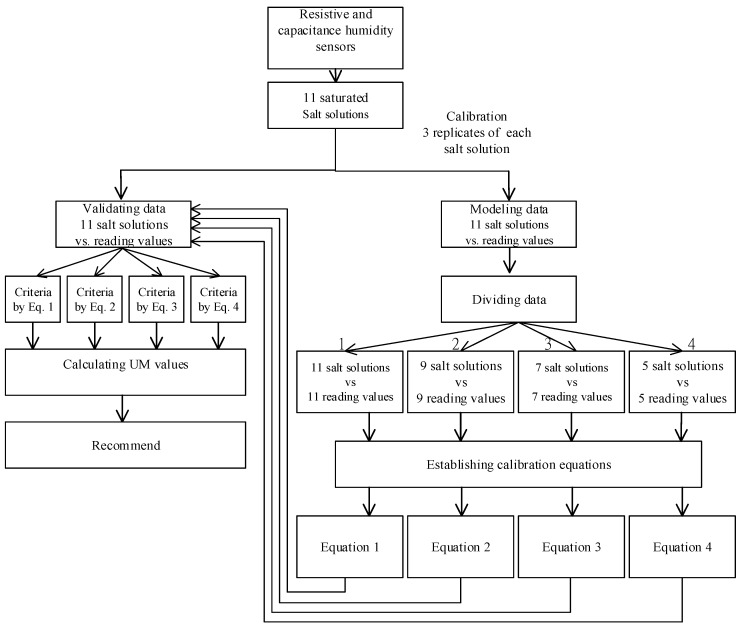
The experimental design and flowchart of data analysis.

The experimental design and flow chart for the data analysis is shown in Figure 1.

The relationship between the standard humidity and the sensor reading values was established as the calibration equation.

This study used the inverse method. The standard humidity is the dependent (y_i_) and the sensor reading values are the independent variables (x_i_) [17].

The form of the linear regression equation is:

Y = b_0_ + b_1_ X (1)

where b_0_ and b_1_ are constants.

The form of the higher-order polynomial equation is:

Y = c_0_ + c_1_X + c_2_X^2^ + c_3_X^3^ +…+c_k_X^k^ (2)

where c_0_, c_1_ to c_k_ are constants.

### 2.5. Different Calibration Points

To model the calibration equations, the data for four different salt solutions was used, as listed in Table 3.

Case 1: The data set is for 11 salt solutions and 11 calibration points
Case 2: The data set is for 9 salt solutions and 9 calibration points
Case 3: The data set is for 7 salt solutions and 7 calibration points
Case 4: The data set is for 5 salt solutions and 5 calibration points

For each sensor, four calibration equations were derived using four different calibration points.

### 2.6. Data Analysis

The software, Sigma plot ver.12.2, was used to determine the parameters for the different orders of polynomial equations.

#### 2.6.1. Tests on a Single Regression Coefficient

The criteria to assess the fit of the calibration equations are the coefficient of determination *R*^2^, the estimated standard error of regression *s* and the residual plots.

The coefficient of determination, *R*^2^ is used to evaluate the fit of a calibration equation. However, no standard criterion has been specified [15,16].

The single parameter coefficient was tested using the *t*-test to evaluate the order of polynomial regression equation. The hypotheses are:

(3) $$H_{0}:b_{k}=0$$

(4) $$H_{1}:b_{k}\neq 0$$

The *t*-value is:

(5) $$t=b_{k}/se(b_{k})$$

where $b_{k}$ is the value of the parameter for the polynomial regression equation of the highest order, and *se*($b_{k}$) is the standard error of $b_{k}$.

#### 2.6.2. The Estimated Standard Error of Regression

The estimated standard error of regression *s* is calculated as follows:

(6) $$s={\left(\frac{{\left({\hat{y}}_{2}-y_{i}\right)}^{2}}{n_{1}-p}\right)}^{0.5}$$

where ${\hat{y}}_{i}$ is the predicted valued of the response, ${\hat{y}}_{i}$ is the response, $n_{1}$ is the number of data and *p* is the number of parameters.

The *s* value is the criterion that is used to determine the accuracy of a calibration equations [38]. It is used to assess the accuracy of two types of RH sensors that are calibrated using different saturated salt solutions.

#### 2.6.3. Residual Plots

Residual plots is the quantitative criterion that is used to evaluate the fit of a regression equation. If the regression model is adequate, the data distribution for the residual plot should tend to a horizontal band and is centered at zero. If the regression equation is not accepted, the residual plots exhibit a clear pattern.

For the calibration equation, tests on a single regression coefficient and the residual plots are used to determine the suitability of a calibration equation for RH sensors that are calibrated using different saturated salt solutions. The estimated standard error of the regression equations is then used to determine the accuracy of the calibration equations.

### 2.7. Measurement Uncertainty for Humidity Sensors

The measurement uncertainty for RH sensors using different salt solutions was calculated using International Organization for Standardization, Guide to the Expression of Uncertainty in Measurement (ISO, GUM) [12,13,17].

u_c_^2^ = u^2^x_pred_ + u^2^_temp_ + u^2^_non_ + u^2^_res_ + u^2^_sta_ (7)

where u_c_ is the combined standard uncertainty, ux_pred_ is the uncertainty for the calibration equation, u_temp_ is the uncertainty due to temperature variation, u_non_ is the uncertainty due to nonlinearity, u_res_ is the uncertainty due to resolution, and u_sta_ is the uncertainty of the reference standard for the saturated salt solution.

The uncertainty of x_pred_ is calculated as follows [38]:

(8) uxpred=s1+1n+(y−y¯)2∑(yi2)−(∑yi)2n

where $\bar{y}$ is the average value of the response.

The uncertainty in the value of u_ref_ for the saturated salt solutions is determined using the reference standard for the salt solution. The scale and the uncertainty of these saturated salt solutions are listed in Table 3 that are taken from Greenspan [18] and the Organisation Internationale De Metrologies Legale (OIML) R121 [19]:

(9) $$u_{\mathrm{ref}}={\left(\frac{\sum {\left(u_{ri}\right)}^{2}}{N_{2}}\right)}^{0.5}$$

where $u_{ri}$ is the uncertainty in the humidity for each saturated salt solution and $N_{2}$ is the number of saturated salt solutions that are used for calibration.

The calibration equations use different numbers of saturated salt solutions had its uncertainty. This criterion is used to evaluate the precision of RH sensors.

The accuracy and precision of RH sensors that are calibrated using different saturated salt solutions was determined using the s and u_c_ values. By Equations (7)–(9), the contrast between the number of saturated salt solutions is considered. The greater the number of data points that are used, the smaller is the s value that is calculated by Equation (6). However, this requires more experimental time and cost and the value of u_ref_ may be increased. The uncertainty of each calibration point is different because different saturated salt solutions are used. The optimal number of calibration points were evaluated by accuracy and precision.

## 3. Results and Discussion

### 3.1. The Effect of the Accuracy of Different Calibration Points

#### 3.1.1. THT-B121 Resistive Humidity Sensor

**Figure 2:**
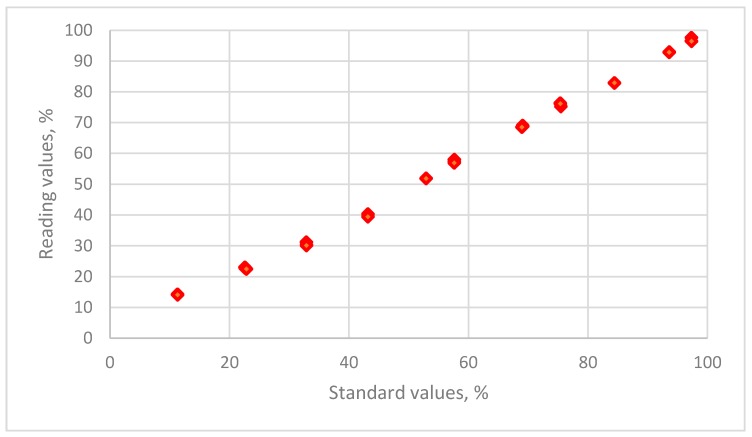
The distribution of the relative humidity data for reading values versus the standard humidity values for THT-B121 resistive humidity sensor using 11 saturated salt solutions (LiCl, CH_3_COOK, MgCl_2_, K_2_CO_3_, Mg(NO_3_)_2_, NaBr, KI, NaCl, KCl, KNO_3_ and K_2_SO_4_).

Calibration equations for resistive sensors using 11 salt solutions:

The distribution of the relative humidity data for the reading values for a resistive sensor is plotted against the standard humidity values that are maintained using 11 saturated salt solutions in Figure 2.

**Figure 3:**
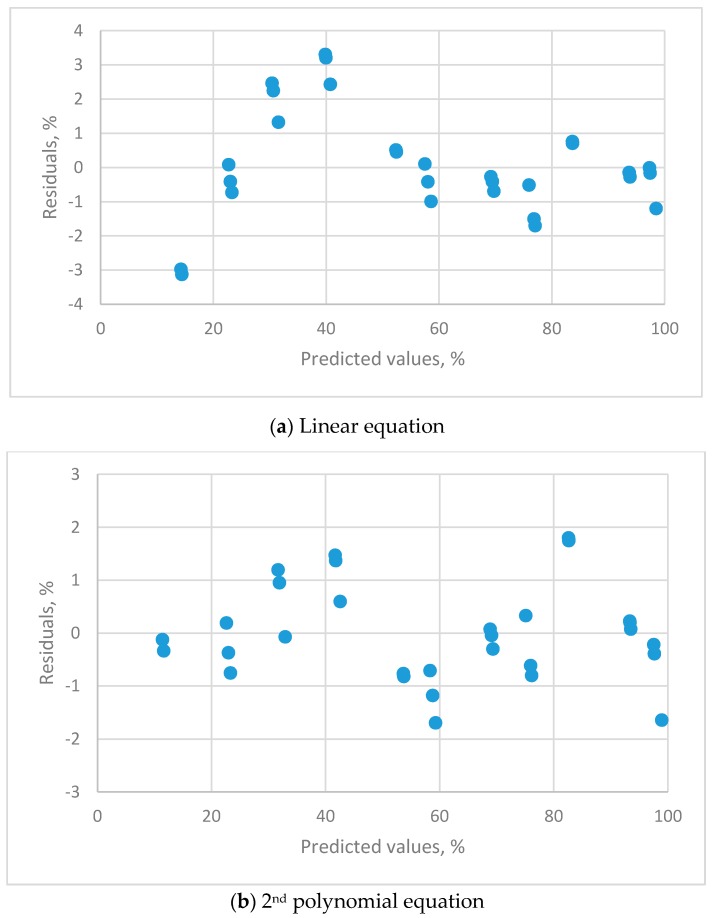

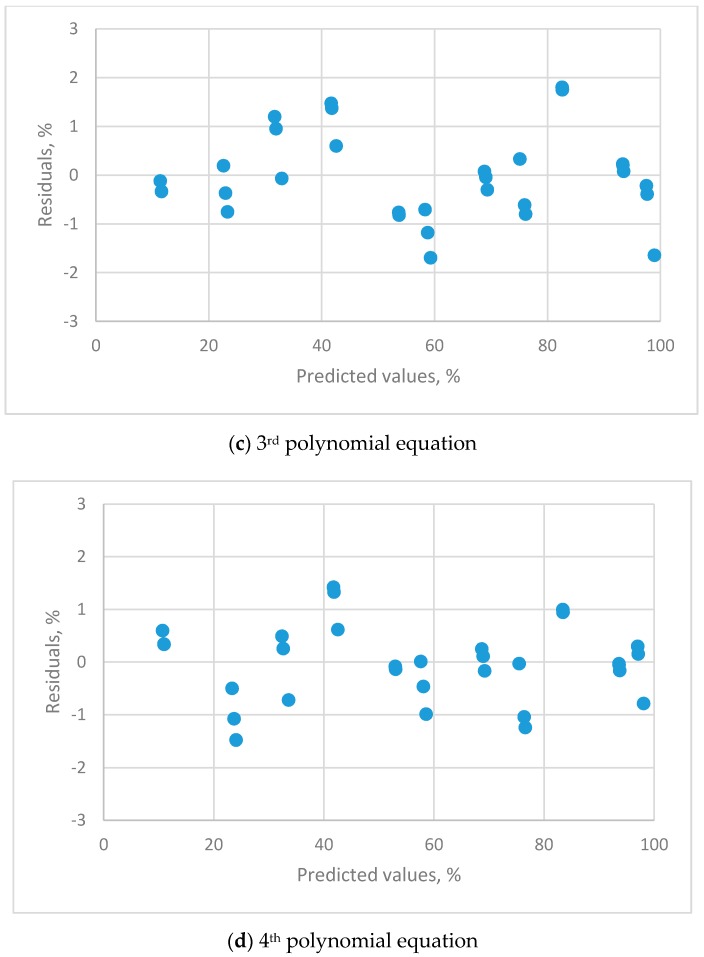
The residual plots for the calibration equations for different orders of polynomial equations for THT-B121 resistive humidity sensor using 11 saturated salt solutions (LiCl, CH_3_COOK, MgCl_2_, K_2_CO_3_, Mg(NO_3_)_2_, NaBr, KI, NaCl, KCl, KNO_3_ and K_2_SO_4_).

The estimated parameters and the evaluation criteria for regression analysis are listed in Table 4. The residual plots for the calibration equations for different orders of polynomial equations are shown in Figure 3.

The linear (Figure 3a), 2nd (Figure 3b) and 3rd (Figure 3c) order polynomial equations all exhibit a systematic distribution of residuals. These equations were not satisfactory for resistive sensors. The distribution of residual plots for the 4th order polynomial equations exhibit a uniform distribution (Figure 3d). The *t*-value for the highest-order parameter (b_4_ = −2.07539 × 10^−6^) was significantly different to zero, so the 4th order polynomial equation is the only adequate calibration equation. The equation is:

y = −20.530298 + 2.805196x − 0.049153x^2^ + 0.000539x^3^ − 2.07539 × 10^−6^x^4^
(s_b_ = 2.5004 s_b_ = 0.2590 s_b_ = 0.0082 s_b_ = 0.00016 s_b_ = 4.770 × 10^−7^
t = −8.2107 t = 11.181 t = −6.005 t = −5.0663 t = −4.3514)
*R*^2^ = 0.992, s = 0.7719

The coefficient of determination, *R*^2^, for the linear, 2nd, 3rd and 4th order polynomial calibration equations are 0.9967, 0.9974, 0.9987 0.9993, respectively. High *R*^2^ values do not give useful information for the specification of an appropriate calibration equation. The estimated values of standard deviation, *s*, is used to define the uncertainty for an inverse calibration equation [35]. The *s* values for the four calibration equations are 1.6098, 1.4612, 0.9820 and 0.7719, respectively. It is seen that an appropriate calibration equation gives a significant reduction in uncertainty.

Calibration equations for resistive sensor using 5 salt solutions:

The estimated parameters and the evaluation criteria for the regression analysis for 5 calibration points for a resistive sensor are listed in Table 5. The residual plots for four calibration equations are shown in Supplementary Materials. Similarly to the regression results for 11 salt solutions, the linear, 2nd and 3rd order polynomial equations all employed a systematic distribution in the residuals plots. These equations are clearly not appropriate calibration equations. For a resistive sensor, the residual plots for the 4th order polynomial equations presented a random distribution.

The *R*^2^ values for the linear, 2nd, 3rd and 4th order polynomial calibration equations are 0.9969, 0.9974, 0.9994 and 0.9998, respectively. However, these higher *R*^2^ values do not provide relevant information about the calibration equations. The *s* values represent the uncertainty of calibration equations. For the linear, 2nd, 3rd and 4th order polynomial calibration equations are 1.8109, 1.7146, 0.7954 and 1.084, respectively. The 4th order polynomial equations is:

y = −19.471802 + 2.743833x − 0.047663x^2^ + 0.0005157x^3^ − 1.93676 × 10^−6^x^4^
(s_b_ = 2.2789 s_b_ = 0.25086 s_b_ = 0.00869 s_b_ = 0.000117 s_b_ = 5.360 × 10^−7^
t = −8.5447 t = 10.9396 t = −5.4849 t = 4.3946 t = −3.6101)
*R*^2^ = 0.991, s = 1.014

The regression results for the 4th order polynomial equations using different calibration points in different salt solutions are listed in Table 6. The results for 9 and 7 calibration points are similar to those for 11 and 5 calibration points.

The *R*^2^ value is used b to evaluate the calibration equations [27,33]. Even the linear calibration equation for this study shows a high *R*^2^ value. However, the estimated error was higher than that for other equations. The residual plots all exhibited a clear pattern distribution so the *R*^2^ value cannot be used as the sole criterion to assess the calibration equation. Betta and Dell’Isola [1] mention *R*^2^, Chi-square and *F*-test to verify the accuracy of a model. This study used *t*-value for a parameter was used as the criterion. This method bases on statistical theory.

#### 3.1.2. HMP 140A Capacitive Humidity Sensor

Calibration equations for a capacitive sensors using 11 salt solutions

**Figure 4:**
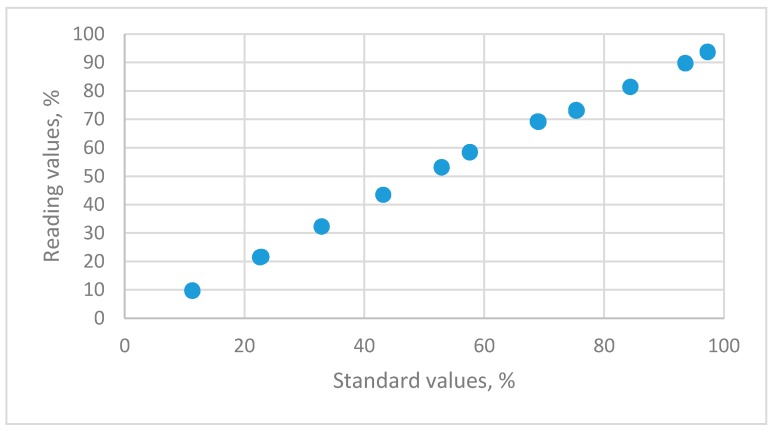
The distributions of relative humidity data for standard humidity values versus the reading values for HMP 140A capacitance humidity sensors using 11 saturated salt solutions (LiCl, CH_3_COOK, MgCl_2_, K_2_CO_3_, Mg(NO_3_)_2_, NaBr, KI, NaCl, KCl, KNO_3_ and K_2_SO_4_).

The relationship between the reading values for a capacitive sensor and the standard humidity values that are maintained using 11 saturated salt solutions is shown in Figure 4.

The estimated parameters and the evaluation criteria for regression analysis are listed in Table 7.

**Figure 5:**
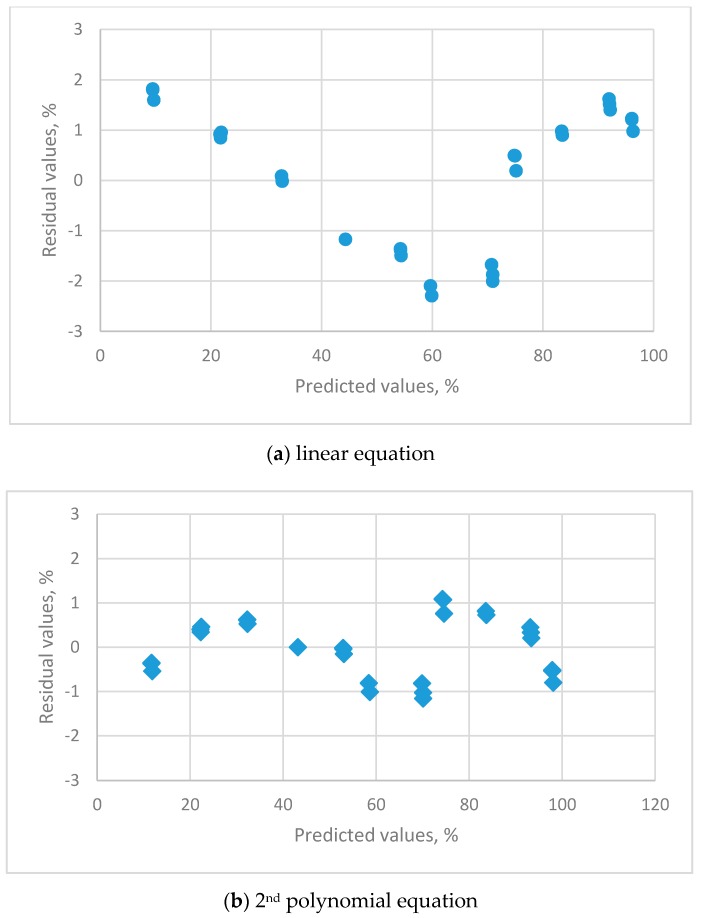
The residual plots for the calibration equations for different orders of polynomial equations for HMP 140A capacitance humidity sensor using 11 saturated salt solutions (LiCl, CH_3_COOK, MgCl_2_, K_2_CO_3_, Mg(NO_3_)_2_, NaBr, KI, NaCl, KCl, KNO_3_ and K_2_SO_4_).

The residual plots for the calibration equations for different orders of polynomial equations are shown in Figure 5.

The linear equation (Figure 5a) exhibited a systematic distribution of residuals. The 2nd (Figure 5b) and 3rd (not presented) order polynomial equations both displayed a uniform distribution. The *t*-value for the 3rd order parameter was not significantly different to zero, so the 2nd order polynomial equation is the appropriate calibration equation and list as follows:

y = 3.479518 + 0.833274x + 0.001867x^2^, *R*^2^ = 0.9994, s = 0.6837
(s_b_ = 0.4805 s_b_ = 0.02028 s_b_ = 0.000187
t = 7.2408 t = 41.098 t = 10.004)

The coefficient of determination, *R*^2^, for the linear and 2nd order polynomial calibration equations are 0.9975 and 0.9994, respectively. The *s* values for the two calibration equations are 1.4002 and 0.6837, respectively. An appropriate calibration equation gives a significant reduction in the estimated error.

Calibration equations for a capacitive sensor using 5 salt solutions

The estimated parameters and the evaluation criteria for the regression analysis for 5 calibration points for a capacitance are listed in Table 8. The residual plots for four calibration equations are shown in Supplementary Materials. Similarly to the regression results for 11 salt solutions, residuals plots for the linear equation exhibit a systematic distribution. Residual plots for the 2nd order polynomial equations presented a random distribution.

The *R*^2^ values for the linear and 2nd order polynomial calibration equations are 0.9981 and 0.9995, respectively. The *s* values for the linear and 2nd order polynomial calibration equations are 1.4386 and 0.7890, respectively. The 2nd order polynomial equations give the smallest estimated errors and listed as follows:

y = 2.9113205 + 0.864217x + 0.0015542x^2^, *R*^2^ = 0.9995, s = 0.7890
(s_b_ = 0.63806 s_b_ = 0.02925 s_b_ = 0.000278
t = 74.5628 t = 29.543 t = 5.5872)

The regression results for the 2nd order polynomial equations using different calibration points in different salt solutions are listed in Table 9. The results of *R*^2^ values for 5, 7, 9 and 11 calibration points are similar. However, the calibration equation for 11 calibration points gives the smallest *s* value.

#### 3.1.3. Evaluation of Accuracy

**Figure 6:**
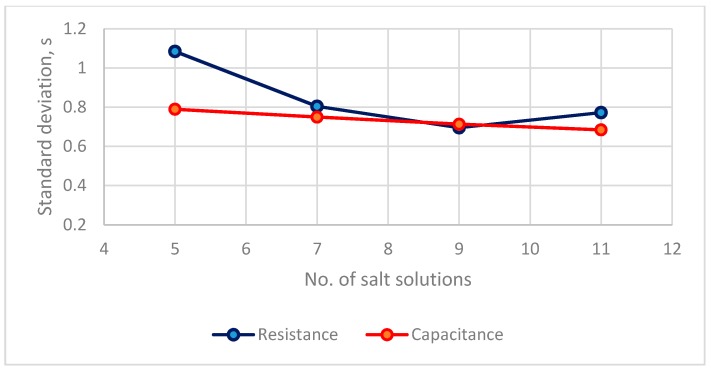
The distribution between numbers of saturated salt solutions and estimated standard errors of calibration equations of two types of RH sensors.

The distribution between the number of saturated salt solutions and the estimated standard error for the calibration equations of two types of RH sensors is in Figure 6. For a resistance sensor, the *s* values of 7, 9, 11 calibration points are <0.8% RH. For a capacitance sensor, the *s* values for four saturated salt solutions are <0.8% RH. The accuracy of these calibration equations is <0.8% for both types of RH sensors. In terms a practical application [20,21], the calibration equation can be established using 7 salt solutions for a resistance sensor and 5 salt solutions for a capacitance sensor.

### 3.2. The Effect of the Precision of Calibration Points

#### 3.2.1. The Measurement Uncertainty for the Two Humidity Sensors

The method that is used to calculate the measurement uncertainty is that of Lu and Chen [17]. Two Types “A” and “B” method are used to evaluate the measurement uncertainty. The Type A standard uncertainty is evaluated by statistical analysis of the experimental data. The Type B standard uncertainty is evaluated using other information that is related to the measurement.

The Type A standard uncertainty for the two types of humidity sensors used the uncertainty for the predicted values from the calibration equations. The Type B standard uncertainty for humidity sensors uses the reference standard, nonlinear and repeatability, resolution and temperature effect. The results for the Type B uncertainty analysis for resistive and capacitive sensors are respectively listed in Table 10 and Table 11. 

**Figure 7:**
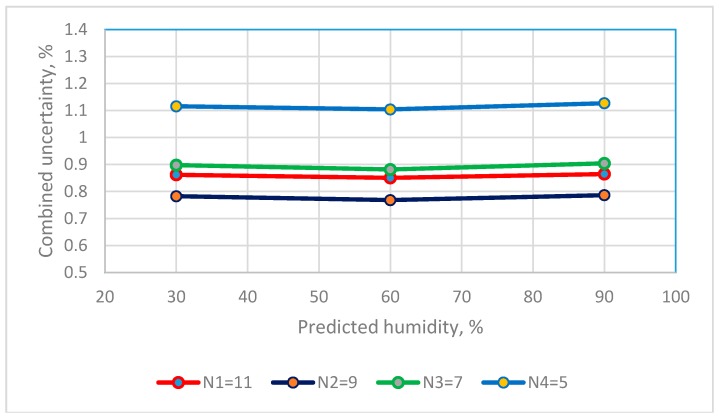
The distribution between numbers of saturated salt solutions and combined uncertainty of resistance RH sensors.

**Figure 8:**
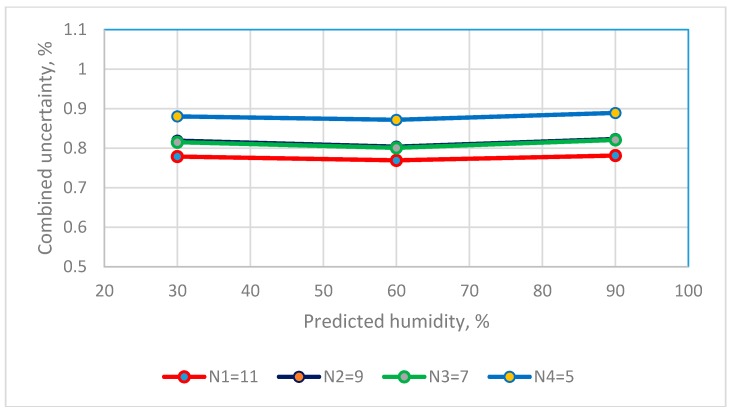
The distribution between numbers of saturated salt solutions and combined uncertainty of capacitance RH sensors.

The Type A standard uncertainty that are calculated using the predicted values for the 4th order polynomial equation for the resistive sensor and the 2nd order polynomial equation for a capacitive sensor are added to give a combined uncertainty using Equation (7). The combined uncertainty for three RH observations for the two humidity sensors using calibration equations that use different calibration points are in Figure 7 and Figure 8.

#### 3.2.2. The Precision of the Two Types of RH Sensors

The combined uncertainty is the criterion that is used to determine the precision of the sensors.

The values for the combined uncertainty for the resistive sensor at a RH of 30%, 60% and 90% are 0.8618%, 0.8506% and 0.8647% for the calibration equation that uses 11 calibration points, and 1.1155%, 1.1040% and 1.1271% for the calibration equation that uses 5 calibration points. The calibration equation that uses 9 calibration points gives the smallest u_c_ values. The combined uncertainty for 7, 9 and 11 calibration points is <1.0% RH.

The values for the combined uncertainty for a capacitive sensor at a RH of 30%, 60% and 90% are 0.7787%, 0.7690% and 0.7813% for the calibration equation that uses 11 calibration points and 0.8803%, 0.8717% and 0.8890% for the calibration equation that uses 5 calibration points. The combined uncertainty for 5, 7, 9 and 11 calibration points is <0.9% RH. In terms of practical applications, this performance is sufficient for industrial applications [20,21].

The accuracy and precision are 0.80% and 0.90% RH for a resistance RH sensor that uses 7 calibration points and 0.70% and 0.90% RH for a capacitance RH sensors that uses 5 calibration points.

### 3.3. Discussion

The number of calibration points that are required for sensors represents a compromise between the ideal number of calibration points and the time and cost of the calibration. The criterion that Betta [1] used to determine the optimal number of points used the ratio of the standard deviation of the regression coefficients (s_bj_) to the established standard error of regression (s).

Accuracy and precision are the most important criteria for sensors so this study uses both values. Using statistical theory, the best calibration equation is determined using the *t*-value for the highest-order parameter and the residual plots. The estimated standard errors for the regression equation are then used to determine the accuracy of the sensors. The combined uncertainty considered the uncertainty of reference materials, the uncertainty for the predicted values and other B type sources. The combined uncertainties for the calibration equations for different numbers of calibration points using different saturated salt solutions are the criteria that are used to evaluate the precision of sensors.

Two types of electric RH sensors were calibrated in this study. Some calibration works, such as those for temperature and pressure sensors, are calibrated by an equal spacing of calibration points. The RH reference environments are maintained using different saturated salt solutions.

It is seen that the optimum number of calibration points that is required to calibrate a resistive humidity sensors involves 7 saturated salt solutions (LiCl, MgCl_2_, K_2_CO_3_, NaBr, NaCl, KCI and K_2_SO_4_), so seven points are specified. Five saturated salt solutions (LiCl, MgCl_2_, NaBr, NaCl and K_2_SO_4)_ are specified for a capacitive humidity sensor. Considering factors that influence the choice of salts, such as price, toxicity and rules for disposal, the choice of these salt solutions is suitable.

The calibration equations key to measurement performance. This study determines that te 4th order polynomial equation is the adequate equation for the resistive humidity sensor and the 2nd order polynomial equation is the optimum equation for the capacitive humidity sensor. The accuracy of the calibration equations is 0.8% RH for a resistive humidity sensor that uses 7 calibration points and 0.7% RH for a capacitance humidity sensor that uses 5 calibration points. The precision is less than 1.0% RH for the resistive sensor and less than 0.9% RH for the capacitive sensor.

The method that is used in this study applicable to other sensors.

## 4. Conclusions

In this study, two types of electric RH sensors were used to illustrate the method for the specification of the optimum number of calibration points. The standard RH environments are maintained using different saturated salt solutions. The theory of regression analysis is applied. The best calibration equation is determined in terms of the *t*-value of the highest-order parameter and the residual plots. The estimated standard errors for the regression equation are the criteria that are used to determine the accuracy of sensors. The combined uncertainty involves the uncertainty for the reference materials, the uncertainty in the predicted values and other B type sources. The combined uncertainties for the calibration equations for different number of calibration points using different saturated salt solutions are the criteria that are used to evaluate the precision of the sensors.

The calibration equations are key to good measurement performance. This study determines that the 4th order polynomial equation is the adequate equation for the resistive humidity sensor and the 2nd order polynomial equation is the best equation for the capacitive humidity sensor. The accuracy of the calibration equations is 0.8% RH for a resistive humidity sensor that uses 7 calibration points and 0.7% RH for a capacitance humidity sensor using 5 calibration points. The precision is less than 1.0% RH for the resistive sensor and less than 0.9% RH for the capacitive sensor.

The method to determine the number of the calibration points used in this study is applicable to other sensors.

## Figures and Tables

**Table 1:** The selection of saturated salt solutions that are used to calibrate humidity sensors.

Salt Solutions	OIMI [19]	Lake [27]	Wadso [28]	Duvernoy [29]	Belhadj [30]	JMS [31]	JISC [32]	CMA [33]	Delta [34]	OMEGA [35]	TA [36]	Vaisala [37]
**LiBr**								*				
**LiCl**	*		*	*		*		*	*	*	*	*
**CH_3_COOK**	*							*		*	*	
**MgCl_2_·GH_2_O**	*		*	*		*	*	*	*	*	*	*
**K_2_CO_3_**	*			*	*	*		*		*	*	
**Mg(NO_3_)_2_**		*	*		*	*		*		*	*	
**NaBr**	*			*		*					*	
**KI**	*	*						*				
**SrCl_2_**											*	
**NaCl**	*	*	*	*	*	*	*	*	*	*	*	*
**(NH_4_)_2_SO_4_**								*				
**KCl**	*	*		*		*	*	*		*	*	
**KNO_3_**					*	*	*			*		
**K_2_SO_4_**	*	*		*	*	*		*		*		*

Note: OIML, The Organisation Internationale De Metrologies Legale.

**Table 2:** The specifications of two humidity sensors.

	Resistive Sensor	Capacitive Sensor
Model 1	THT-B121	HMP 140A
Sensing element	Macro-molecule HPR-MQ	HUMICAP
Operating range	0–60 °C	0–50 °C
Measuring range	10–99% RH	0–100%
Nonlinear and repeatability	±0.25% RH	±0.2% RH
ResolutionTemperature effect	0.1% RH (relative humidity)none	0.1% RH0.005%/°C

**Table 3:** The Calibration points for saturated salt solutions to establish the calibration equations.

Salt Solutions	(n_1_ = 11) Case 1	(n_2_ = 9) Case 2	(n_3_ = 7) Case 3	(n_4_ = 5) Case 4	u_c_
LiCl	*	*	*	*	0.27
CH_3_COOK	*				0.32
MgCl_2_	*	*	*	*	0.16
K_2_CO_3_	*	*	*		0.39
Mg(NO_3_)_2_	*	*			0.22
NaBr	*	*	*	*	0.40
KI	*	*			0.24
NaCl	*	*	*	*	0.12
KCl	*	*	*		0.26
KNO_3_	*				0.55
K_2_SO_4_	*	*	*	*	0.45

Note: u_c_ values were obtained from Greenspan [18] and The Organisation Internationale De Metrologies Legale (OIML) R121 [19].

**Table 4:** Estimated parameters and evaluation criteria for the linear and several polynomial equations for THT-B121 resistive sensor using 11 salt solutions.

	Linear	2nd Order	3nd Order	4th Order
b_0_	0.028672	−2.74999	−11.0702	−20.5303
b_1_	1.008985	1.13766	1.780025	2.805196
b_2_		−0.0011437	−0.01432	−0.0491534
b_3_			7.81681 × 10^−5^	5.39281 × 10^−4^
b_4_				−2.07539 × 10^−6^
*R* ^2^	0.9967	0.9974	0.9987	0.9993
s	1.6098	1.4612	0.982	0.7719
Residual plots	clear pattern	clear pattern	clear pattern	uniform distribution

**Table 5:** Estimated parameters and evaluation criteria for the linear and several polynomial equations for THT-B121 resistive sensors using 5 salt solutions.

	Linear	2nd Order	3nd Order	4th Order
b_0_	−0.970118	−3.1191770	−12.201481	−19.471802
b_1_	1.0155235	1.12632754	1.8869907	2.743833
b_2_		−0.001007316	−0.01685101	−0.04766345
b_3_			9.34623 × 10^−5^	5.15689 × 10^−4^
b_4_				−1.93676 × 10^−6^
*R* ^2^	0.9969	0.9974	0.9994	0.9991
s	1.8109	1.7146	0.7984	1.084
Residual plots	clear pattern	clear pattern	clear pattern	uniform distribution

**Table 6:** Estimated parameters and evaluation criteria for the 4th order polynomial equations for THT-B121 resistive sensors using four different calibration points.

	Case 1 (n_1_ = 11)	Case 2 (n_2_ = 9)	Case 3 (n_3_ = 7)	Case 4 (n_4_ = 5)
b_0_	−20.530297	−23.41845561	−23.904948	−19.4718019
b_1_	2.8051965	3.5861653	3.243023015	2.743832845
b_2_	−0.04915334	−0.06230766	−0.06426625	−0.047663446
b_3_	5.39281 × 10^−4^	7.0951 × 10^−4^	7.34202 × 10^−4^	5.15689 × 10^−4^
b_4_	−2.07539 × 10^−6^	−2.81734 × 10^−6^	−2.92042 × 10^−6^	−1.93676 × 10^−6^
*R* ^2^	0.9993	0.9994	0.9994	0.9991
s	0.7719	0.6951	0.8039	1.084

**Table 7:** Estimated parameters and evaluation criteria for the linear and polynomial equations for HMP 140A capacitive sensor using 11 salt solutions.

	Linear	2nd Order
b_0_	−0.414520	3.479518
b_1_	1.031003	0.833274
b_2_		0.00186718
*R* ^2^	0.9975	0.9994
s	1.4002	0.6837
Residual plots	clear pattern	Uniform distribution

**Table 8:** Estimated parameters and evaluation criteria for the linear and polynomial equations for HMP 140A capacitive sensor using 5 salt solutions.

	**Linear**	**2nd Order**
b_0_	0.226512	2.911321
b_1_	1.023088	0.814217
b_2_		0.00155423
*R* ^2^	0.9981	0.9995
s	1.4386	0.7890
Residual plots	clear pattern	Uniform distribution

**Table 9:** Estimated parameters and evaluation criteria for the 2nd order polynomial equations for HMP 140A capacitive sensors using four different calibration points.

	Case 1 (n_1_ = 11)	Case 2 (n_2_ = 9)	Case 3 (n_3_ = 7)	Case 4 (n_4_ = 5)
b_0_	3.479580	3.156891	2.871078	2.9113205
b_1_	0.833274	0.844157	0.862302	0.8142171
b_2_	0.00186718	0.00176878	0.00161775	0.00155423
*R* ^2^	0.9975	0.9992	0.9994	0.9995
s	0.6837	0.7127	0.7490	0.7890

**Table 10:** The Type B uncertainty analysis for resistive humidity sensor.

Description	Estimate Value (%)	Standard Uncertainty u(x), (%)
Reference standard, U_ref_		N_1_ = 11, u_ref_ = 0.3311 N_1_ = 9, u_ref_ = 0.2983 N_1_ = 7, u_ref_ = 0.3151 N_1_ = 5, u_ref_ = 0.3084
Non-linear and repeatability, U_non_	±0.3	0.00866
Resolution, U_res_	0.1	0.00290
The combined standard uncertainty of Type B = 0.1926		

**Table 11:** The Type B uncertainty analysis for capacitive humidity sensor.

Description	Estimate Value (%)	Standard Uncertainty u(x), (%)
Reference standard, U_ref_		N_1_ = 11, u_ref_ = 0.3311 N_1_ = 9, u_ref_ = 0.2983 N_1_ = 7, u_ref_ = 0.3151 N_1_ = 5, u_ref_ = 0.3084
Nonlinear and repeatability, U_non_	±0.1	0.0058
Resolution, U_res_	±0.1	0.0029
Temperature effect, U_temp_	±0.005	0.0043
The combined standard uncertainty of Type B = 0.1924		

## Supplementary Materials

The following are available online at https://www.mdpi.com/1424-8220/19/5/1213/s1. The residual plots for the calibration equations for different orders of polynomial equations for resistive humidity sensor using 5 saturated salt solutions (LiCl, MgCl_2_, NaBr, NaCl and K_2_SO_4_). The residual plots for the calibration equations for different orders of polynomial equations for capacitance humidity sensor using 5 saturated salt solutions (LiCl, MgCl_2_, NaBr, NaCl and K_2_SO_4_).
