# Supplementary material for: Determination of Optimal Measurement Points for Calibration Equations—Examples by RH Sensors
Source: Sensors (Basel). 2019 Mar 9;19(5):1213. doi: 10.3390/s19051213 (PMC6427136; doi:10.3390/s19051213)
Supplement: Supplementary file 1 [file sensors-19-01213-s001.pdf]

Appendix. The residual plots for the calibration equations for different orders of polynomial equations for resistive humidity sensor using 5 saturated salt solutions (LiCl, $MgCl_2$, NaBr, NaCl and K_2SO_4).

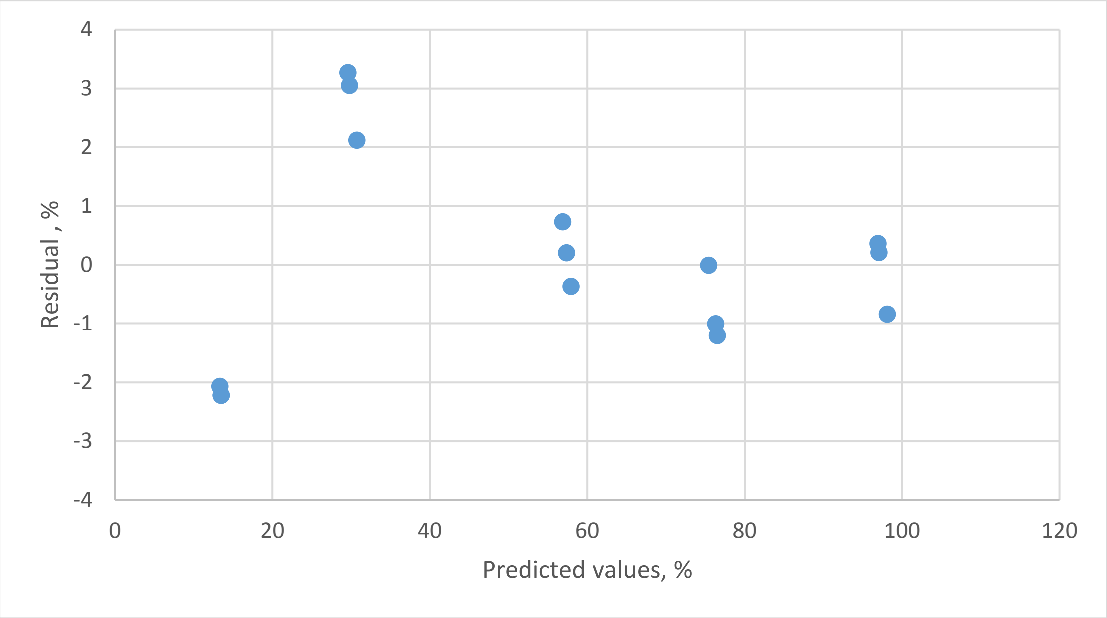

a. linear equation

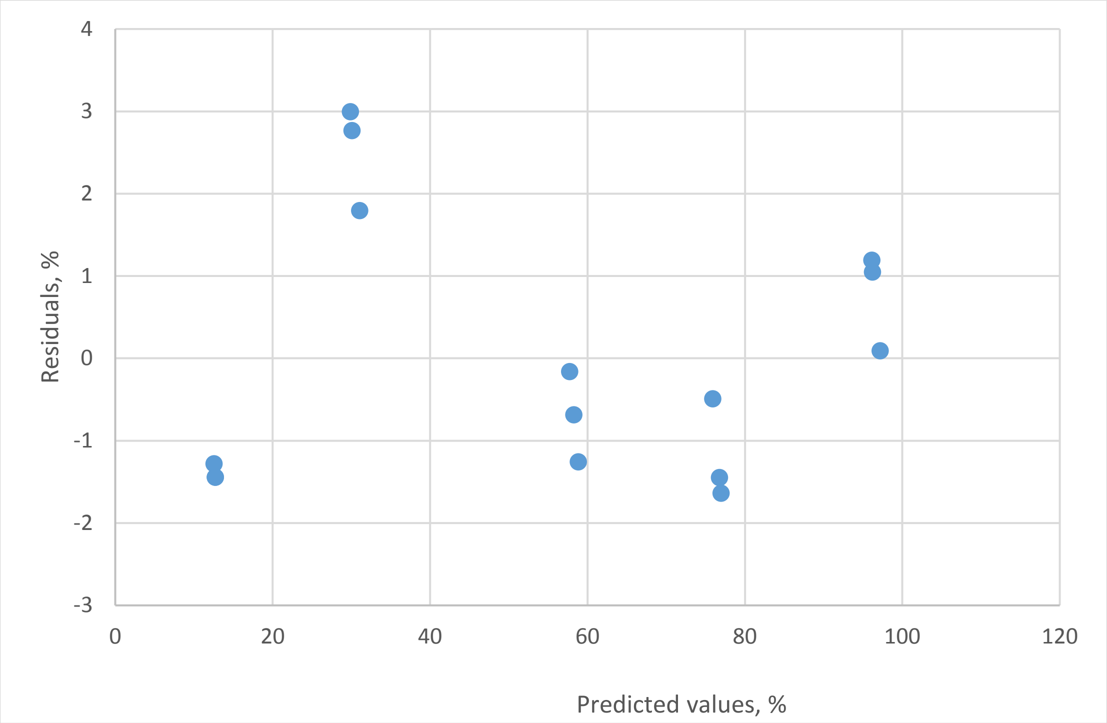

b. 2nd polynomial equation

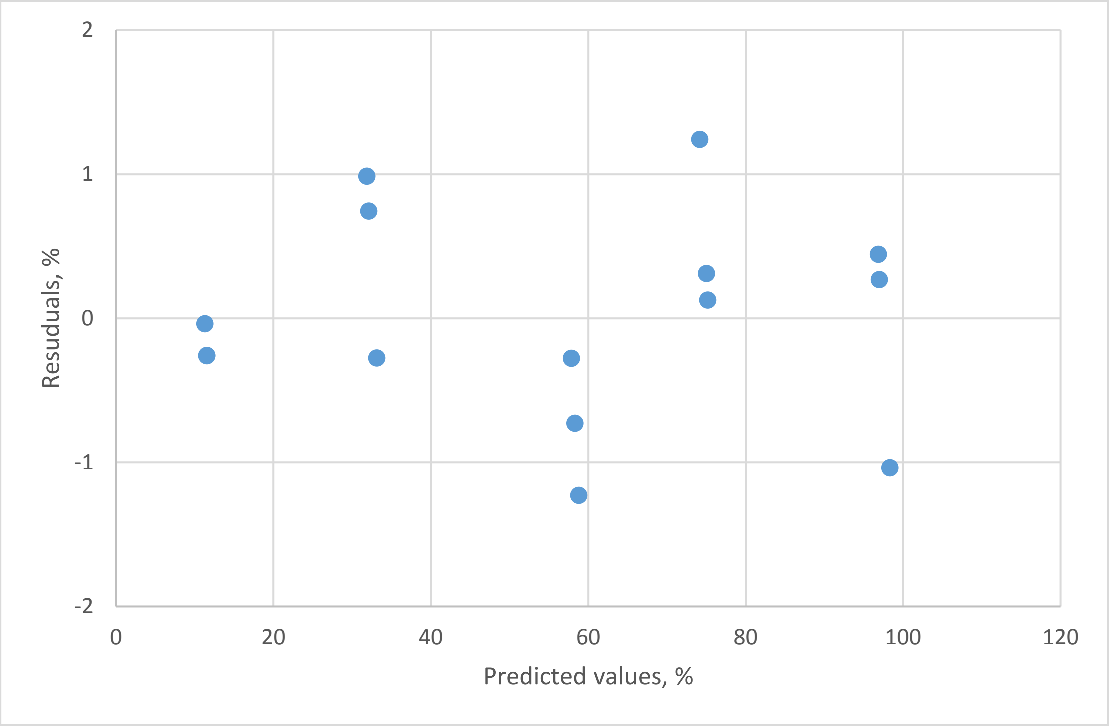

c. 3rd polynomial equation

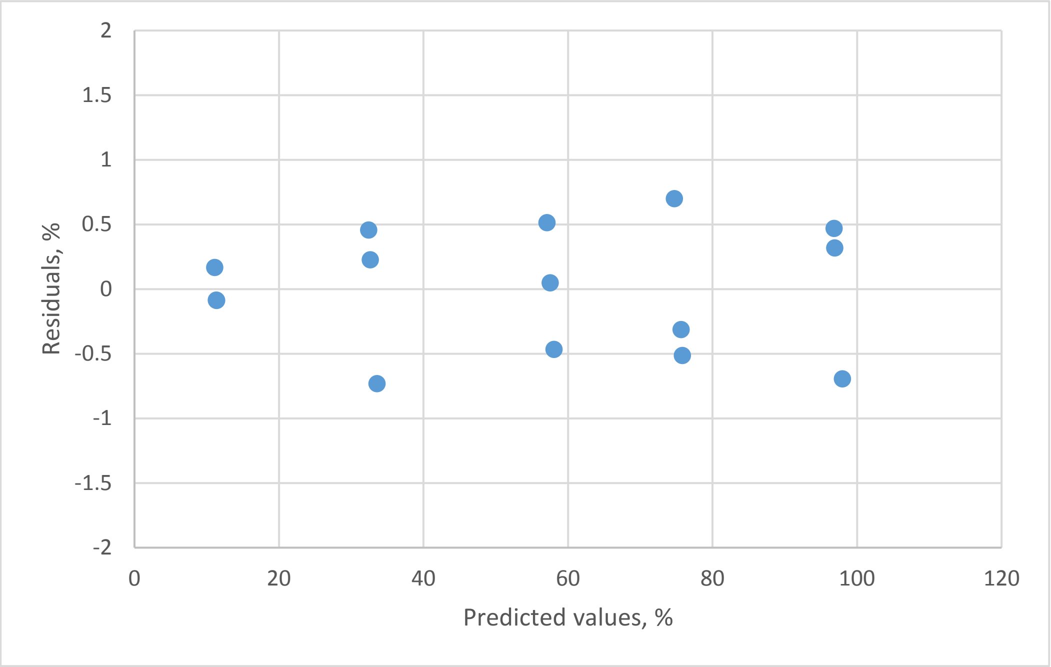

d. 4th polynomial equation

Appendix. The residual plots for the calibration equations for different orders of polynomial equations for capacitance humidity sensor using 5 saturated salt solutions (LiCl, $MgCl_2$, NaBr, NaCl and K_2SO_4).

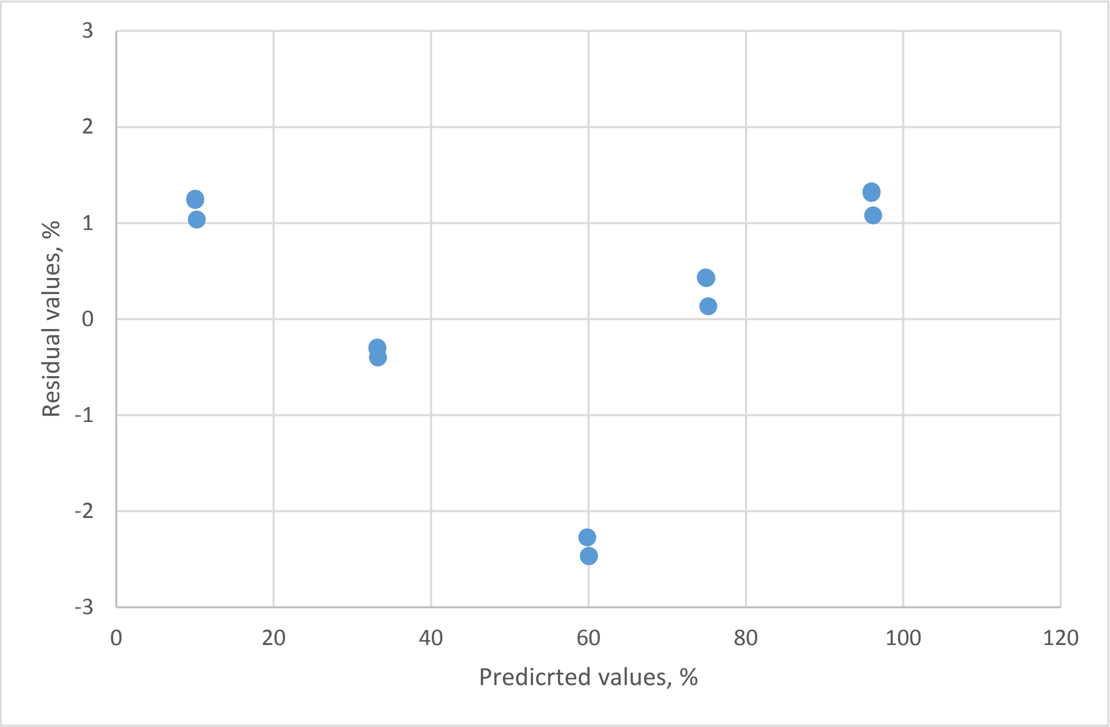

a. Linear equation

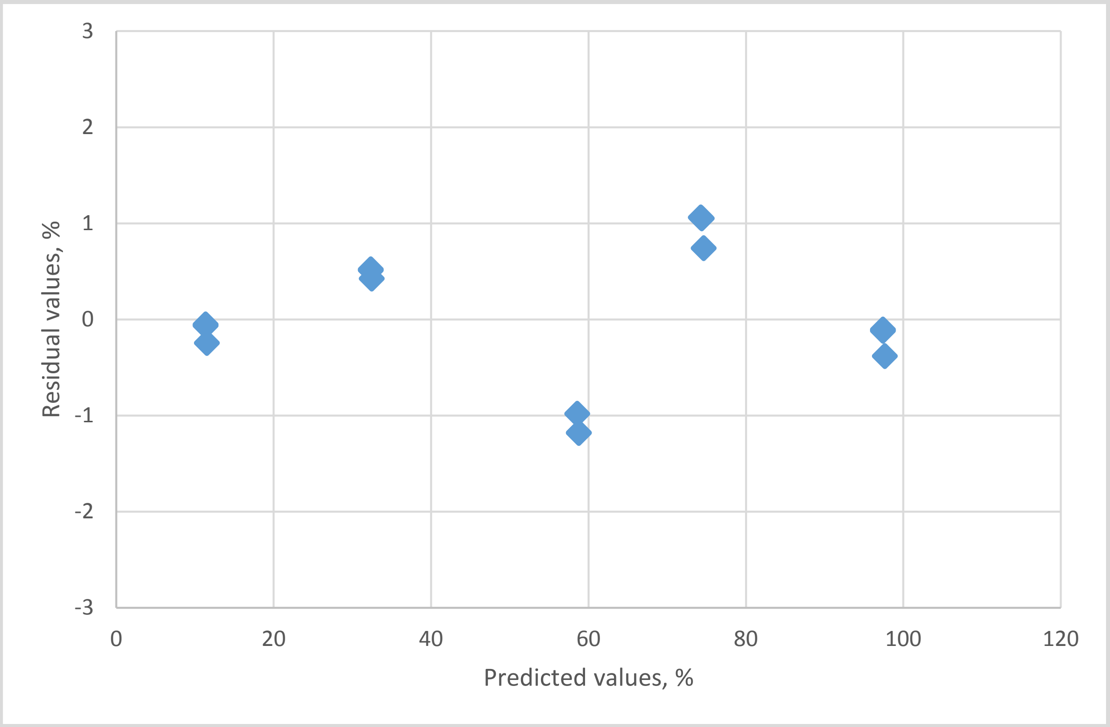

b. 2nd polynomial equation
